# Enhancement of persistent currents and magnetic fields in a two dimensional quantum ring

**DOI:** 10.1038/s41598-023-42417-2

**Published:** 2023-09-19

**Authors:** Vinod Prasad, Monika Arora, Varsha

**Affiliations:** 1 Department of Physics, Swami Shraddhanand College, University of Delhi, Delhi, 110036 India; 2 Department of Mathematics, Miranda House, University of Delhi, Delhi, 110007 India; 3 Department of Physics and Astrophysics, University of Delhi, Delhi, 110007 India; 4 Department of Physics, Kalindi College, University of Delhi, Delhi, 110008 India

**Keywords:** Semiconductors, Spintronics

## Abstract

We present the study of the SiGe quantum ring (QR) modeled by an anharmonic axially symmetric potential with a centrifugal core in the effective mass approximation. We show how the femtosecond laser pulses (FLPs) can be used efficiently for controlling the induced current and magnetic field. We have compared the strength of induced currents and magnetic fields with and without pulsed laser which shows a substantial change. The spin-orbit interaction (SOI) and Zeeman energy show a massive impact on the generation and enhancement of these induced current and magnetic fields. These induced currents and magnetic fields have many applications in interdisciplinary areas. We have shown that the SOI presence with the FLP fields while competing with the confinement strength lowers the strength of the induced current and field.

## Introduction

Spintronics is a promising field for researchers working in the nanomaterial field around the globe as it uses the properties of the electrons to store and process the information (optoelectronic devices)^1, 2^. The investigation of two-dimensional(2D) nanostructures such as quantum ring(QR), disc has gained the extensive interest both theoretically and experimentally^3–7^. The spin study has applications in a wide range from quantum computing, quantum computers, memory storage devices, communication, spin transistors, and spin filters, etc.^8–14^.

The spin-orbit interaction (SOI) splits the energy levels of the nanostructure in spin-up and spin-down states^15^. The interaction of orbital angular momentum and spin magnetic moment causes this splitting. The SOI can be caused either by lack of inversion symmetry in nanostructure crystals which is known as Dresselhaus spin-orbit interaction(DSOI)^16^ or due to structural inversion symmetry in nanostructure crystals which is known as Rashba spin-orbit interaction(RSOI)^17, 18^. The spin of the charge carriers can be manipulated by external electric fields, magnetic fields, doping, size of the nanostructure, electromagnetic field interaction among other ways, as this changes the strength of the SOI which in turn alters the physical and optical properties of the nanostructure^19–22^.

Datta and Das presented a spin transistor based on the impact of spin precessions due to the RSOI^23^. The polarized laser pulses are used to induce persistent current which generates induced magnetic field which is a well-known study in literature^24–28^. In 2003, Splettstoesser et al.^29^ studied the impact of spin-orbit coupling (SOC) on persistent currents in mesoscopic conducting rings. They have shown that the different flux dependences of persistent charge and spin currents are a unique signature of spin-orbit coupling affecting the electronic structure of the ring. Abiague and Berakdar^30^ shown that by adjusting the time delay and intensity of the light pulses in nanoscopic and mesoscopic ring structures, the amplitude and direction of the induced currents may be controlled. Electric ring currents and magnetic fields induced by short intense circularly polarized $\pi$ laser pulses in atomic orbitals are detailed by the Barth and Manz^31^. They found the largest ring current and fields for 2$p_{\pm }$ orbitals and they reported that the currents and associated induced magnetic fields are much larger than those generated by static magnetic fields. Moskalenko and Berakdar^32^ studied the nonequilibrium dynamics in a mesoscopic graphene ring excited by picoseconds shaped electromagnetic pulses and predicted an ultrafast buildup of charge polarization, currents, and orbital magnetization. Charge-current generation in atomic systems induced by optical vortices is investigated by K$\ddot{o}$ksal and Berakdar^33^. The S-matrix method is used to investigate spin transport across a new geometry of double quantum ring(QR) in the presence of the RSOI by Dehghan et al^34^. By using an unpolarized incoming spin current, they shown that their new geometry of double QRs can be used as a flawless spin-filtering nano-device.

Prasad et al.^35^ studied the persistent currents and the corresponding induced magnetic field at the nucleus of spherically confined hydrogenic atoms in the presence of Hulth$\acute{e}$n potential. They reported that the persistent currents and induced magnetic field are profoundly affected by the potential parameters, spherical confinement parameter, etc. The anharmonic axially symmetric potential with a centrifugal core has a finite radius to width ratio that can be constantly modified from strictly one-dimensional to completely two-dimensional. Kovalev and Chaplik^36^ studied the magnetoplasma oscillation frequencies of 2D electrons in a QR using a model with a finite ring width, allowing for a precise solution for the single-particle spectrum. They have shown that the case under consideration depends on the magnetic flux with frequency and amplitude modulations. The period of oscillations as a function of the magnetic flux and their amplitude are both dependent on the magnetic field strength. In reference^37^, Santander et al. studied the optical properties of an exciton in a finite radius-to-width ratio QR where they presented the analytical solution when the electron-hole interaction is short-ranged. They have shown that the Aharonov-Bohm oscillations of the ground state of the exciton decreases with increasing width. The linear and nonlinear optical properties are calculated using exact solutions for the two-dimensional motion of a conduction band electron in a disc-shaped GaAs quantum dot under the influence of an external magnetic field and parabolic and inverse square confining potentials by Duque et al.^38^. The authors have shown that the contribution of the nonlinear third-order factors in both absorption and relative correction to the refractive index increases dramatically as the strength of the inverse square potential function increases. Xie^39^ studied photoionization cross-section in a two-dimensional QR using an anharmonic axially symmetric potential with a centrifugal core. The author confirmed that the resonant peak of the photoionization cross-section demonstrates the Aharonov-Bohm effect as a function of the magnetic flux with the Aharonov-Bohm effect.

The optical response of 2D QR(Tan- Inkson ring) in presence of RSOI is studied by Gumber et al.^40^. In their work, the authors showed that the RSOI produced avoided crossing regions at finite magnetic fields where the optical absorption is suppressed drastically and the ring becomes transparent to incident light. Later the authors have studied the spin transport in a Rashba-coupled 2D QR analytically^41^ where they gave the expression for spin-polarized conductance of the ring. Therefore, this potential has a wide application in 2D QRs and analytical solutions are possible. Lumb et al.^42^ studied the generation of charge currents and hence induced magnetic field in a spherically bound hydrogen atom in the presence of Hulth$\acute{e}$n potential by an ultrafast right circularly polarized laser pulse. Magnetic pulses(of the order of femtosecond) which have many biomedical applications are produced by the laser pulse employed^43^. Recently, the class of Yukawa potential is explored for persistent current, magnetic susceptibility, and thermal properties in the non-relativistic regime, in the presence of external magnetic and Aharonov-Bohm fields, using the asymptotic iteration approach by Edet et al.^44^.

Till now, the strength of the observed induced currents and fields is very low. For example, Cho et al.^45^, reported a circular current of 0.36 nA in a double quantum dot system. With some heating difficulties, Lidar and Thywissen have reported a peak magnetic field of 10 mT generated by a limited array of several tens of superconducting Nb nanowires^46^. Pershin and Piermarocchi demonstrated that utilising phase-locked infrared laser pulses, the localised magnetic field in an isolated semiconducting QR can be on the order of 3 mT, and that the magnetic field can be adjusted by the laser pulses^47^. In their another work, the authors studied a quantum wire with two QRs attached at the ends geometry^48^. They have chosen this specific geometry as it provides the optimal condition for exploiting the broken time-reversal symmetry in the circularly polarized electromagnetic field which gave nearly 8pA induced current. This method of current generation could be explored in cabon nanotubes loops. Nandipati and Vendrell^49, 50^ studied generation of electronic ring currents under vibronic coupling effects by circularly polarized light with and without presence of dynamical Jahn-Teller effect. They have reported induced current of the order of approx. 6mA. The charge currents and induced magnetizations of hydrogen atoms with noncentral interaction, submerged in a plasma environment, are studied theoretically under the impact of spherical enveloping by Bahar^51^. The effects of plasma shielding, spherical confinement, and the non-central effect on the production and management of charge-currents and induced magnetizations are examined in depth in this work.

These remarkable studies explain the importance of the induced currents, and magnetic fields which motivated us for our present work in the manuscript. Here we propose the study of induced current, and induced magnetic field for 2D SiGe QR modeled with an anharmonic axially symmetric potential with a centrifugal core with the presence of magnetic field considering the SOI. In our system we are able to obtain few Tesla order of induced magnetic field and induced current of the order of 0.01 A. Spectral characteristics are greatly modified by the laser pulse, which has a direct impact on transport processes. By adjusting the laser pulse from the outside, we can effectively control the induced current, and the induced local magnetic field. To our best knowledge, these properties under SOC in presence of a magnetic field have not been studied for this system.

The paper is structured as follows: The section method explains the relevant details about the system under consideration, and it is followed by the results section, where the findings of our study are presented in detail, followed by the discussion section giving the outcome of the work. Finally, the conclusion section gives the main findings of the work.

## Method

We consider a 2D QR in the presence of magnetic field (applied along z direction) modeled using an anharmonic axially symmetric potential with a centrifugal core written as

1 $$\begin{aligned} V(r)=C_1 r^{2} + C_2 r^{-2} \end{aligned}$$

The Hamiltonian of the system in an external magnetic field presence is given as

2 $$\begin{aligned} H_0=\frac{\left( p+e\vec {A}\right) ^2 }{2m^*} + V(r) \end{aligned}$$

where $\vec {A}$ is the vector potential given as $\vec {A}=\frac{\vec {B}\times \vec {r}}{2}$ and $m^*$ is the effective mass of the electron. This time independent Schr$\ddot{o}$dinger equation follows

3 $$\begin{aligned} H_0 \varphi _{nl}(r, \phi )=\epsilon _{nl} \varphi _{nl}(r, \phi ) \end{aligned}$$

where $\varphi _{nl}(r, \phi )$ is the unperturbed wavefunction and $\epsilon _{nl}$ are the eigen-energies corresponding to *n* principal quantum number and *l* magnetic quantum number. We consider

4 $$\begin{aligned} \varphi _{nl}(r, \phi )= \frac{e^{i\;l\phi }}{\sqrt{2\pi }} \frac{R_{nl}}{\sqrt{r}} \end{aligned}$$

The radial part of the Schr$\ddot{o}$dinger equation is given as

5 $$\begin{aligned} \left[ \frac{-\hbar ^{2}}{2 m^*}\frac{\partial ^2}{\partial r^2}+\frac{l\,B\hbar }{2 m^*}+\frac{\left( l^2-\frac{1}{4}\right) \hbar ^{2}}{2\,m^* \,r^2}+ \frac{B^2\,r^2}{8\,m^*} + V(r) \right] R_{nl}=\,\epsilon _{nl} R_{nl} \end{aligned}$$

The magnetic field term is acting as repulsive potential term. If $r_0$ is the confining radius, then depending upon the strength of confinement, we can divide interaction into three categories namely (a) weak confinement ($r_0$ is larger than the Bohr atomic radius, here potential is behaving like harmonic potential), (b) strong confinement ($r_0$ is smaller than the Bohr atomic radius, here potential is behaving like inverse square potential), and (c) intermediate confinement ($r_0$ is comparable to Bohr atomic radius), the region where we will see the interplay of $r^2$ and $r^{-2}$ terms. The weak confinement region is more like a free system so the analytic solutions are proposed for the same by Duque et al.^38^. The analytic solution^38^ for energy eigenvalues and eigenfunctions for the Eq. (5) is given as

6 $$\begin{aligned}{} & {} E_{nl}(r, \phi )\,=\, \left( 2n+1+\sqrt{\frac{2m^* C_2}{\hbar ^2}+l^2}\right) \hbar \sqrt{\frac{2C_1}{m^*}+ \left( \frac{eB}{2m^*}\right) ^2} + \frac{l\hbar e B}{2m^*} \end{aligned}$$

7 $$\begin{aligned}{} & {} \varphi _{nl}(r, \phi )\,=\,\sqrt{\frac{{n!}}{\pi \Gamma \left( 2s_l + n+ \frac{1}{2}\right) \eta ^{4s_l +1}}} \frac{1}{\sqrt{r}} r^{2s_l} e^{\frac{-r^2}{2\eta ^2}} L^{2s_l-\frac{1}{2}}_n \left( \frac{r^2}{\eta ^2}\right) e^{i l \phi } \end{aligned}$$

where $$\eta = \frac{\hbar }{m^* \sqrt{\frac{2C_1}{m^*}+\frac{e^2B^2}{4{m^*}^2}}} , \,\,n=\frac{\epsilon _{nl}-l\hbar eB/2m^*}{4 \hbar \sqrt{\frac{2C_1}{m^*}+\frac{e^2B^2}{4{m^*}^2}}} - s_l - \frac{1}{4}$$,  $$s_l = \frac{1}{4}+ \sqrt{ \frac{(2m^*C_2/\hbar ^2) + l^2}{4}}$$

$L^b_n(x)$ is associated laguerre polynomial, and $\Gamma (c)$ is Euler gamma function .

In strong and intermediate confinement region, the quantum effects are in play. In strong confinement region, the potential acts as inverse square potential and in intermediate region, the harmonic and inverse square terms of the potential have antagonistic effects, therefore the Eq. (5) can’t be solved analytically. We solved the equation using finite difference numerical method using nine point stencil for $l\ne 0$. For $l=0$  the  nine-point  finite  difference  method  does  not  give  accurate convergence  in  the  results, therefore  we  solve  $l=0$  cases using a  linear  variational  approach  with  the  basis  set  taken as wavefunctions  of  a  free  particle  in  a  circle^52^.

Considering the RSOI and DSOI, the Hamiltonian can be written as

8 $$\begin{aligned} H\,=\,H_0 \,I + H_B \,I+ H_R + H_D \end{aligned}$$

Here I is the identity matrix of order $2\times 2$, $H_B = \frac{g\, \mu _{B}\,B\,\sigma _{z}}{2}$ is Zeeman energy term(+ for spin-down and − for spin-up state), g denotes the Landé g factor, $\mu _{B}$ is Bohr magneton, and $\sigma =(\sigma _x, \,\sigma _y, \,\sigma _z)$ are the Pauli spin matrices. The Hamiltonian RSOI and Hamiltonian DSOI are denoted by $H_R$ and $H_D$ respectively. The expressions for $H_R$ and $H_D$ are given by^15, 53^

9 $$\begin{aligned} H_R\,= & {} \, i \alpha \left( -sin\phi \sigma _x + cos\phi \sigma _y \right) \frac{\partial }{\partial r} - \frac{i \alpha \left( sin\phi \sigma _y + cos\phi \sigma _x \right) }{r} \left( \frac{\partial }{\partial \phi } + \frac{i\,B\pi \,r^2}{\phi _0} \right) \end{aligned}$$

10 $$\begin{aligned} H_D\,= & {} \,i \beta \left( -cos\phi \sigma _x + sin\phi \sigma _y \right) \frac{\partial }{\partial r} + \frac{i \beta \left( cos\phi \sigma _y + sin\phi \sigma _x \right) }{r} \left( \frac{\partial }{\partial \phi } + \frac{i\,B\pi \,r^2}{\phi _0} \right) \end{aligned}$$

Here $\phi _0\,=\, \frac{h}{e}$ is the quantum flux. $\alpha$ and $\beta$ are the Rashba and Dresselhaus coupling constants respectively. Due to presence of the Pauli spin matrices, the resulting Hamiltonian is of the form

$$\begin{aligned} H = \begin{pmatrix} H_{11} &{} H_{12} \\ H_{21} &{} H_{22}\\ \end{pmatrix} \end{aligned}$$

The wave function for total Hamiltonian H is a linear combination of the spin up and the spin down states, given as

$$\begin{aligned} \psi _{nl} = \begin{pmatrix} \psi _{nl_\uparrow } \\ \psi _{nl_\downarrow } \\ \end{pmatrix} \end{aligned}$$

where

11 $$\begin{aligned} \psi _{nl_\uparrow } = \Sigma _{nl} C_{nl\uparrow }\, \varphi _{nl}(r, \phi ) \end{aligned}$$

and

12 $$\begin{aligned} \psi _{nl_\downarrow } = \Sigma _{nl} C_{nl\downarrow }\, \varphi _{nl}(r, \phi ) \end{aligned}$$

Here $\uparrow$ signifies spin-up state and $\downarrow$ signify spin-down state. Therefore, the Schr$\ddot{o}$dinger equation takes the matrix form as $H \psi _{nl} = E_{nl} \psi _{nl}$ where $E_{nl}$ is the new energies after spin splitting.

The matter-laser interaction study is crucial for understanding the response of the system. Here we have considered interaction with a FLP of duration $t_p \,=$ 4 fs with amplitude $E_0$ and angular frequency $\omega$ polarized in the $\hat{\xi }$ which is expressed as

13 $$\begin{aligned} \vec {E}(t) = \hat{\xi }E_0 f(t) cos (\omega t) \end{aligned}$$

The shape of the pulse envelop *f*(*t*) is given by

$$\begin{aligned} f(t)=\left\{ \begin{array}{l l l} sin^2 (\frac{\pi t}{t_p}) &{},&{} 0\le t \le t_p\\ 0 &{},&{} otherwise. \end{array} \right. \end{aligned}$$

The time-dependent Schr$\ddot{o}$dinger equation of the system is given by

14 $$\begin{aligned} i \frac{\partial \Theta (t)}{\partial t } = (H - V(t) )\Theta (t) \end{aligned}$$

The interaction potential is given as

15 $$\begin{aligned} V(t) = r E_0 f(t) cos (\theta ) \, cos (\omega t) \end{aligned}$$

where $\theta$ is the angle between the polarization direction of the laser pulse and $\hat{r}$.The wavefunction $\Theta (t)$ can be expressed as a linear combination of $\psi _{nl}$ as $\psi _{nl}$ forms a complete set of orthonormal basis. The dressed wavefunction of the system is given as

16 $$\begin{aligned} \Theta (t) = \Sigma _n \Sigma _l a_{nl} (t) \psi _{nl} \end{aligned}$$

## Results

We have calculated results for a 2D $Si_{1-x}Ge_{x}$ QR where *x* is the concentration of *Ge*. In this study, we have used $x=0.3$. The parameters used for $Si_{0.7}Ge_{0.3}/Si$ QR are as follows: $\sigma =2.8\times 10^{22}m^{-3}$, $n_{r}=3.55$, $\varepsilon =13.05$, $\Gamma _{\textrm{fi}}=1/0.38$ $ps^{-1}$^56^, and $B=10T$ unless mentioned otherwise.

Without the pulsed laser field, the current density is time independent and is in $\phi$ directional only i.e. azimuthal component of current density only. To showcase the order of the induced current and magnetic field without pulsed laser (to be discussed later in the text).

The current density in presence of SOI and Zeeman energy without the laser field is defined as^15^

17 $$\begin{aligned} \vec {j_{nl}}(r,\phi )\,=\, \frac{i\hbar }{2m^*} \left( \psi _{nl} \nabla {\psi ^*_{nl} } - {\psi ^*_{nl} } \nabla {\psi _{nl} } \right) \,=\, \frac{l\hbar }{m^* r}|\psi _{nl}|^2 \hat{\phi } + \frac{e}{m^*} \psi ^*_{nl} A \psi _{nl} \hat{\phi } \end{aligned}$$

As we have mentioned earlier the presence of pulsed laser lead to dressed wavefunction $\Theta (t)$ which is a linear combination of the individual states for different *n* and *l* with time-dependent coefficients. $\Theta (t)$ is solution of time dependent Schrödinger equation (Eq. 14) explained in “Method” section. Hence it gives rise to a time dependent $I_{\phi }$, $I_r$, and $B_{\phi }$. These induced currents and magnetic fields are of short duration which is shown later on.

Short-duration current and magnetic fields are of utmost importance in biomedical applications. For example, such magnetic fields and current pulses have been used in heart valves^54^ successfully and many other applications^43, 55^.

When the system is exposed to the short pulsed laser then the probability current density of the system can be calculated as^52^

18 $$\begin{aligned} \vec {j}(r,\phi ,t)\,=\, \frac{i\hbar }{2m^*} \left( \Theta (t) \nabla {\Theta ^* (t) } - {\Theta ^* (t) } \nabla {\Theta (t) } \right) \end{aligned}$$

This current density has both radial and azimuthal components giving rise to current and magnetic fields in both radial as well as azimuthal directions. Using expressions for Eq. (5) explained in “Method” section, we obtain the currents and magnetic fields as shown in figures.

While studying the concept of the induced current, it is crucial to understand the variation of current density as it defines the probability of finding the electron in a region of space with time. The wavefunction is defined in terms of a dressed state due to the presence of a magnetic field and a laser field, which modifies the fluctuation of current density in the radial and in the azimuthal direction. As an example, we observe positive and negative peaks in current density, as the states are not pure but a linear combination of all the states. The behavior of current density is strongly dependent on the type of laser pulse employed, the frequency and intensity of the laser field, and the strength of the magnetic field.

**Figure 1:**
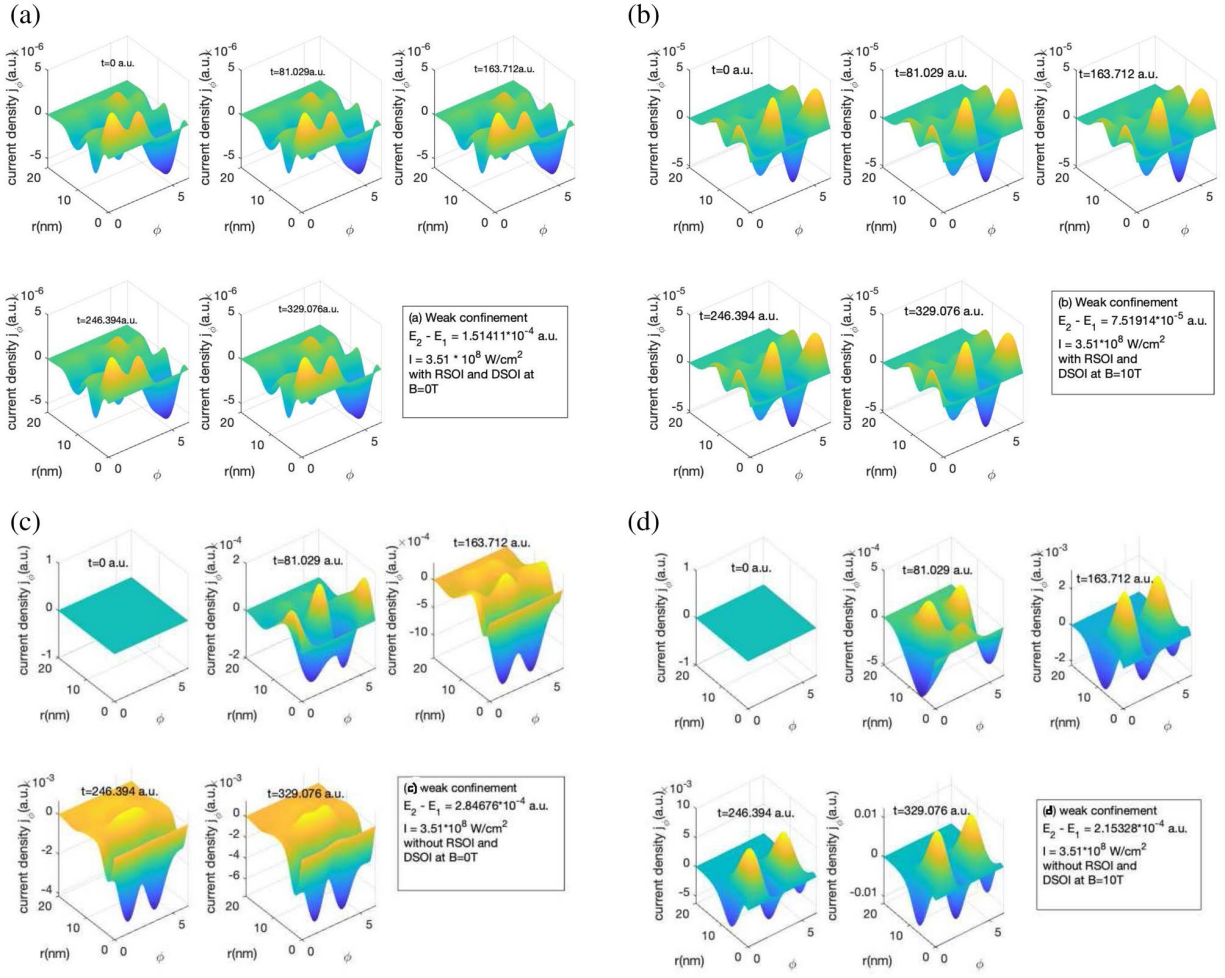
Variation of $j_{\phi }$ with the *r* and $\phi$ for a short laser pulse of $t_p$ = 4 fs, I=$10^{-4}$ a.u., $\hbar \omega = (E_2 - E_1)$ for weak confinement region (**a**) in the absence of magnetic field with RSOI and DSOI with $\alpha = 5 meV nm$ and $\beta = 10 meV nm$ (**b**) in the presence of magnetic field strength of 10T with RSOI and DSOI with $\alpha = 5 meV nm$ and $\beta = 10 meV nm$ (**c**) in the absence of magnetic field without RSOI and DSOI (**d**) in the presence of magnetic field strength of 10T without RSOI and DSOI for time (i) t = 0 a.u., (ii) t = 81.029 a.u., (iii) t = 163.712 a.u., (iv) t = 246.394a.u., (v) t = 329.076 a.u.

The current density leads to the current generation which in turn induces a magnetic field whose magnitude is calculated by using Biot-Savart law. The azimuthal component of current density is shown in Fig. 1 for a short laser pulse ($t_p$ = 4 fs) for weak confinement region. The intensity of laser pulse is taken to be $10^{-4}$ a.u. The energy of the laser pulse is taken to be as the difference of the ground and the first excited state.

We have both positive and negative peaks which shows the mixed behavior of states. With the application of the magnetic field, we observe that there is an increase in the amplitude of the current density. The competition of confinement effect and Zeeman energy decides the net value which with an increase in confinement strength, the impact of the magnetic field on current density will grow. This is because a loosely confined system has a large volume which leads to lowering of energy levels compared to the tightly bound system. Here it is interesting to note that due to the presence of the SOI, as soon as the system is exposed to the laser pulse, the current density is induced instantly to the maximum. We observe that with an increase in confinement strength (i.e. reducing the confinement radius) the azimuthal induce current density increases and the energy of the system increases as can be seen in Supplementary information section.

It is interesting to note that the SOI lowers the strength of the induced current density. This is accounted as the system is now having dressed wavefunction instead of pure individual states which changes the induced current density and hence current and magnetic fields. As we can see that in weak confinement in Fig. 1a it is $10^{-6}$ a.u. and in Fig. 1c it is $10^{-3}$ a.u. at the end of the pulse. In the presence of magnetic field i.e. in Fig. 1b, the amplitude of induced current density has increased compared to the absence of magnetic field Fig. 1a in weak confinement. The increase in amplitude with the presence of magnetic field is due to the increase in the Zeeman energy term in the Hamiltonian and the linear and quadratic term in Eq. (5) explained in “Method” section.

**Figure 2:**
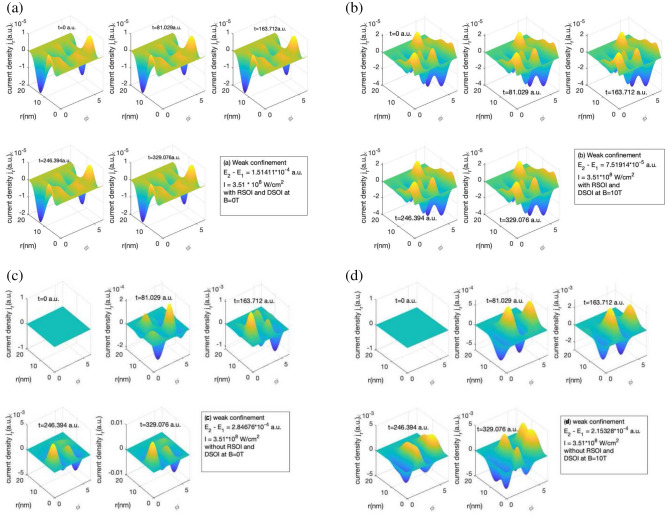
Variation of $j_r$ with the *r* and $\phi$ for a short laser pulse of $t_p$ = 4 fs, I=$10^{-4}$ a.u., $\hbar \omega = (E_2 - E_1)$ for weak confinement region (**a**) in the absence of magnetic field with RSOI and DSOI with $\alpha = 5 meV nm$ and $\beta = 10 meV nm$ (**b**) in the presence of magnetic field strength of 10T with RSOI and DSOI with $\alpha = 5 meV nm$ and $\beta = 10 meV nm$ (**c**) in the absence of magnetic field without RSOI and DSOI (**d**) in the presence of magnetic field strength of 10T without RSOI and DSOI for time (i) t = 0 a.u., (ii) t = 81.029 a.u., (iii) t = 163.712 a.u., (iv) t = 246.394 a.u., (v) t = 329.076 a.u.

The total induced current density is the sum of radial and azimuthal components, so we have also calculated the radial component of induced current density. Figure 2 shows the radial component of the induce current density with and without RSOI and DSOI for a short laser pulse for weak confinement region. In Fig. 2, we observe that the existence of a magnetic field introduces Zeeman energy into the system, increasing the induced current density. The strength of the radial current density increases with the increase in the confinement strength and magnetic field i.e. in weak confinement $j_r$ is of the order of the $10^{-5}$ a.u., in an intermediate region, it is of the order of the $10^{-4}$ a.u., and in strong confinement region, it is of $10^{-3}$ a.u. order as can be seen from Supplementary Information section. The reason for this is that increasing the confinement’s strength increases the energy and affects the quantum levels’ wavefunction, this is visible in Fig. 2a and b. We observe that due to the presence of the SOI in the system, the current density($j_r$) rises to a maximum as soon as the laser pulse is initiated as we can see from Fig. 2a and b. However, in the absence of RSOI and DSOI, the magnitude of the $j_r$ increases with the duration of the laser pulse, i.e. the strength grows with time as shown in Fig. 2c and d. As the total wavefunction of the system is a linear combination of all the states after the laser pulse interaction, we observe both positive and negative peaks as visible in Fig. 2. The role of magnetic field and confinement is similar to discussed for Fig. 1.

**Figure 3:**
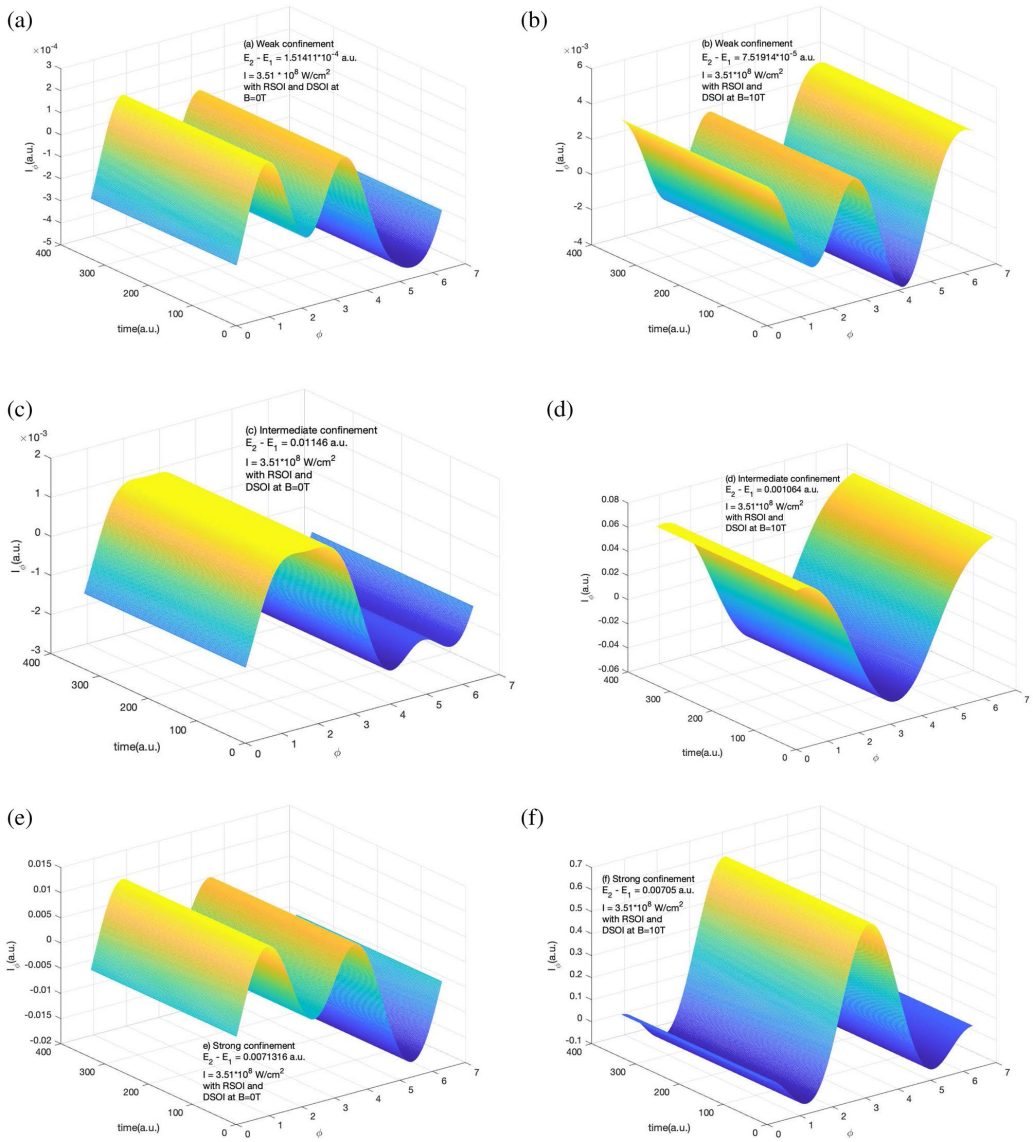
Variation of $I_{\phi }$ in the presence of RSOI and DSOI with the time of the laser pulse(in a.u.) and $\phi$ for a short laser pulse of $t_p$ = 4 fs, I=$10^{-4}$ a.u., $\hbar \omega = (E_2 - E_1)$ (**a**) for weak confinement region in the absence of magnetic field (**b**) for weak confinement region in the presence of magnetic field strength of 10T (**c**) for intermediate confinement region in the absence of magnetic field (**d**) for intermediate confinement region in the presence of magnetic field strength of 10T (**e**) for strong confinement region in the absence of magnetic field (**f**) for strong confinement region in the presence of magnetic field strength of 10T.

The information of induced current density is important as this helps us in understanding the response of induced current in the system along the radial and azimuthal directions. Figure 3 shows the variation of $I_{\phi }$ in the presence of RSOI and DSOI with the time of the laser pulse (in a.u.) and $\phi$ for a short laser pulse as explained in Fig. 1 and Supplementary information section. Here we observe the positive and negative peaks along with ${\phi }$ which is due to the current density peaks which is also visible in induced current density curve in Fig. 1. The presence of a magnetic field increased the magnitude of the induced current as the current density also increases. We observe that with an increase in the confinement strength, the magnitude of the azimuthal component of induced current grows roughly 10 times at B = 10T.

**Figure 4:**
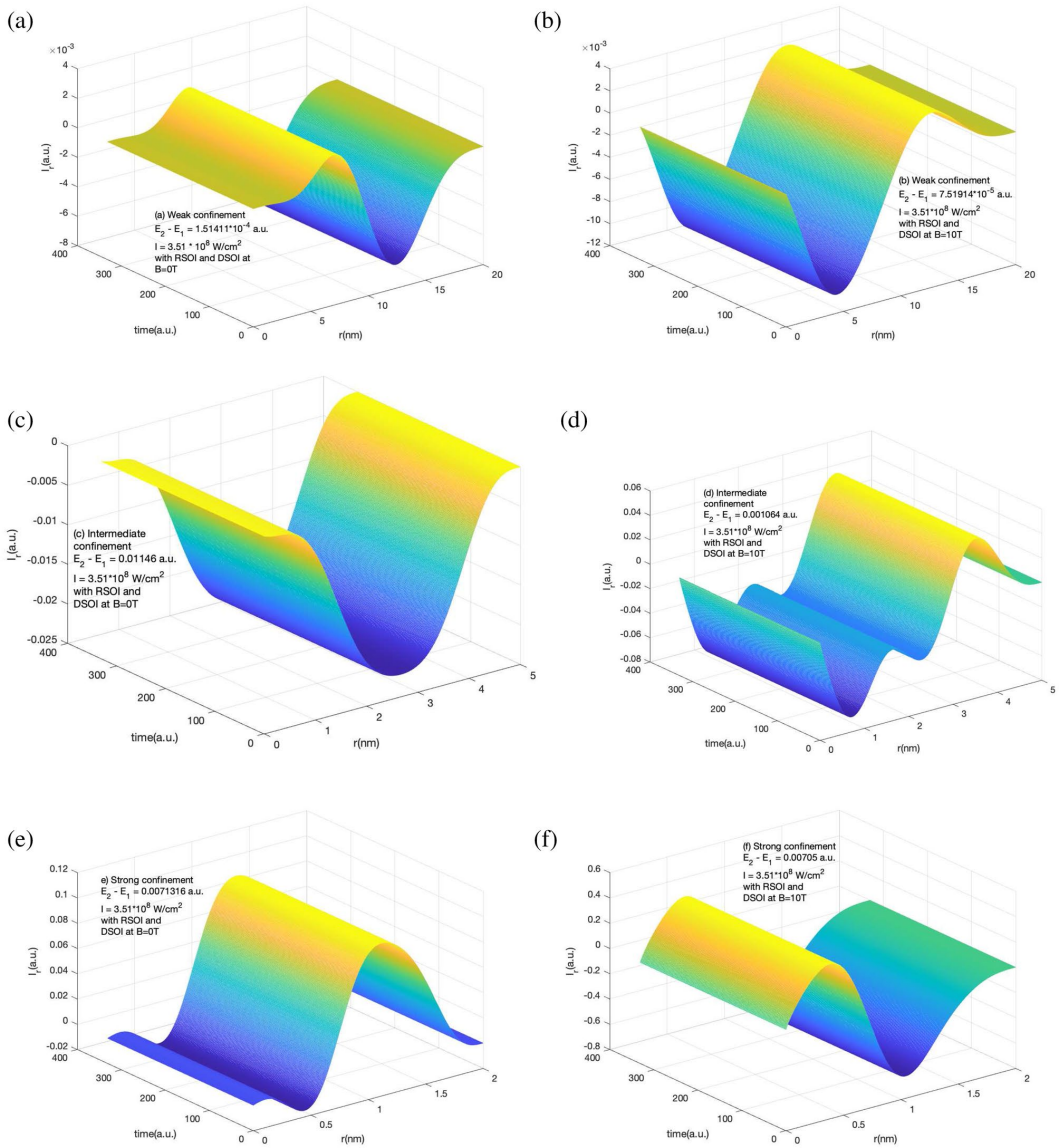
Variation of $I_r$ in the presence of RSOI and DSOI with the time of the laser pulse(in a.u.) and *r* for a short laser pulse of $t_p$ = 4 fs, I = $10^{-4}$ a.u., $\hbar \omega = (E_2 - E_1)$ (**a**) for weak confinement region in the absence of magnetic field (**b**) for weak confinement region in the presence of magnetic field strength of 10 T (**c**) for intermediate confinement region in the absence of magnetic field (**d**) for intermediate confinement region in the presence of magnetic field strength of 10 T (**e**) for strong confinement region in the absence of magnetic field (**f**) for strong confinement region in the presence of magnetic field strength of 10 T.

The corresponding radial component of the induced current is shown in Fig. 4. It shows the variation of $I_r$ in the presence of RSOI and DSOI with the time of the laser pulse(in a.u.) and *r* for a short laser pulse of 4 fs duration of intensity $10^{-4}$ a.u. of frequency equal to the difference of ground and first excited state. The three confinement regions of study are shown i.e. for weak confinement (a) and (b), for intermediate confinement (c) and (d), and for strong confinement (e) and (f) in the absence and presence of magnetic field respectively. Here too, we observe the positive and negative peaks along with *r* and the strength of induced current increases with an increase in the confinement strength. The effect of the magnetic field is dominant in the strong magnetic field. From Figs. 3 and 4, it is clear that due to the presence of SOI in the system, the current induced is constant with time. This is because the maximum current density appears instantly when the laser is exposed to the system as shown in Figs. 1 and 2.

**Figure 5:**
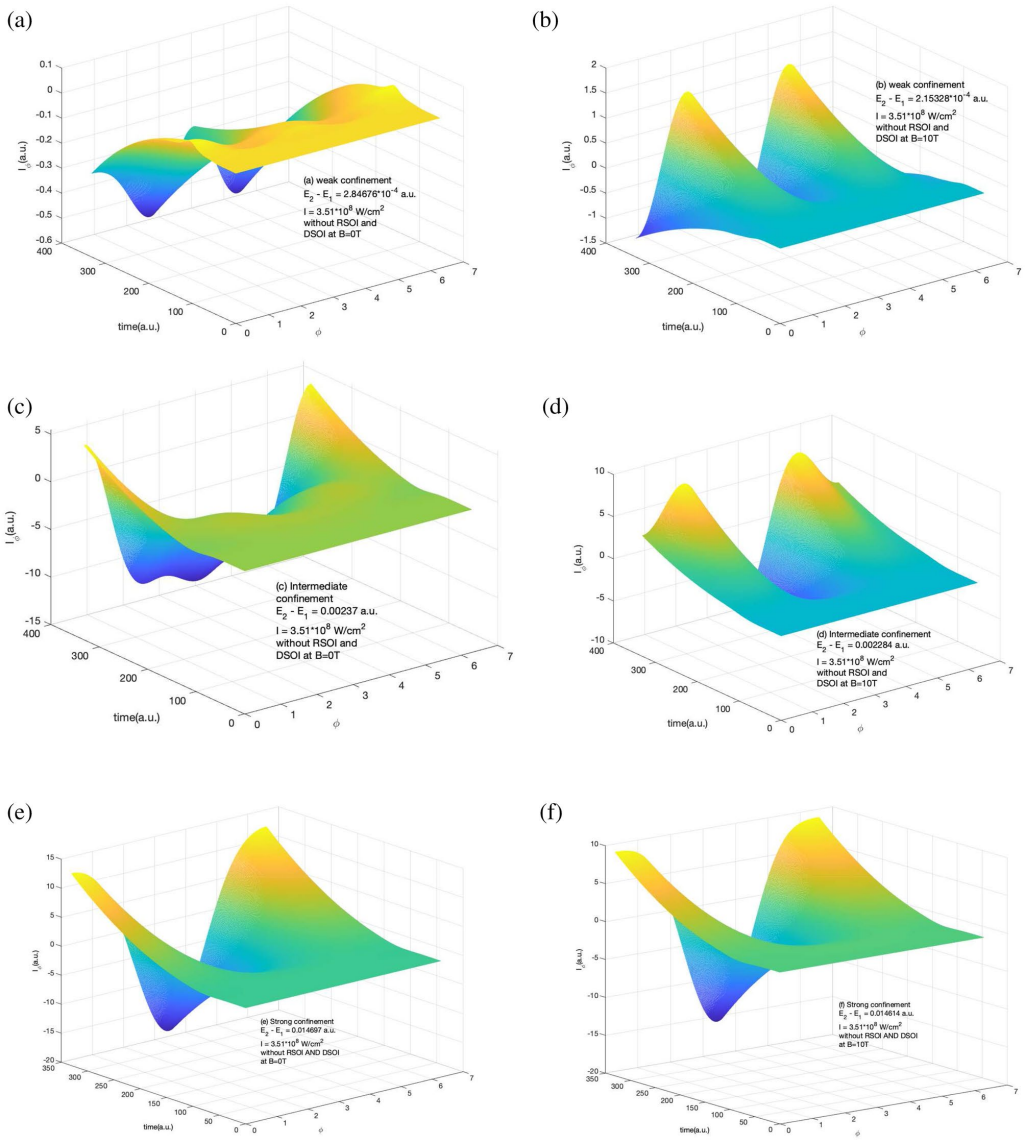
Variation of $I_{\phi }$ in the absence of RSOI and DSOI with the time of the laser pulse (in a.u.) and $\phi$ for a short laser pulse of $t_p$ = 4 fs, I=$10^{-4}$ a.u., $\hbar \omega = (E_2 - E_1)$ (**a**) for weak confinement region in the absence of magnetic field (**b**) for weak confinement region in the presence of magnetic field strength of 10T (**c**) for intermediate confinement region in the absence of magnetic field (**d**) for intermediate confinement region in the presence of magnetic field strength of 10 T (**e**) for strong confinement region in the absence of magnetic field (**f**) for strong confinement region in the presence of magnetic field strength of 10T.

**Figure 6:**
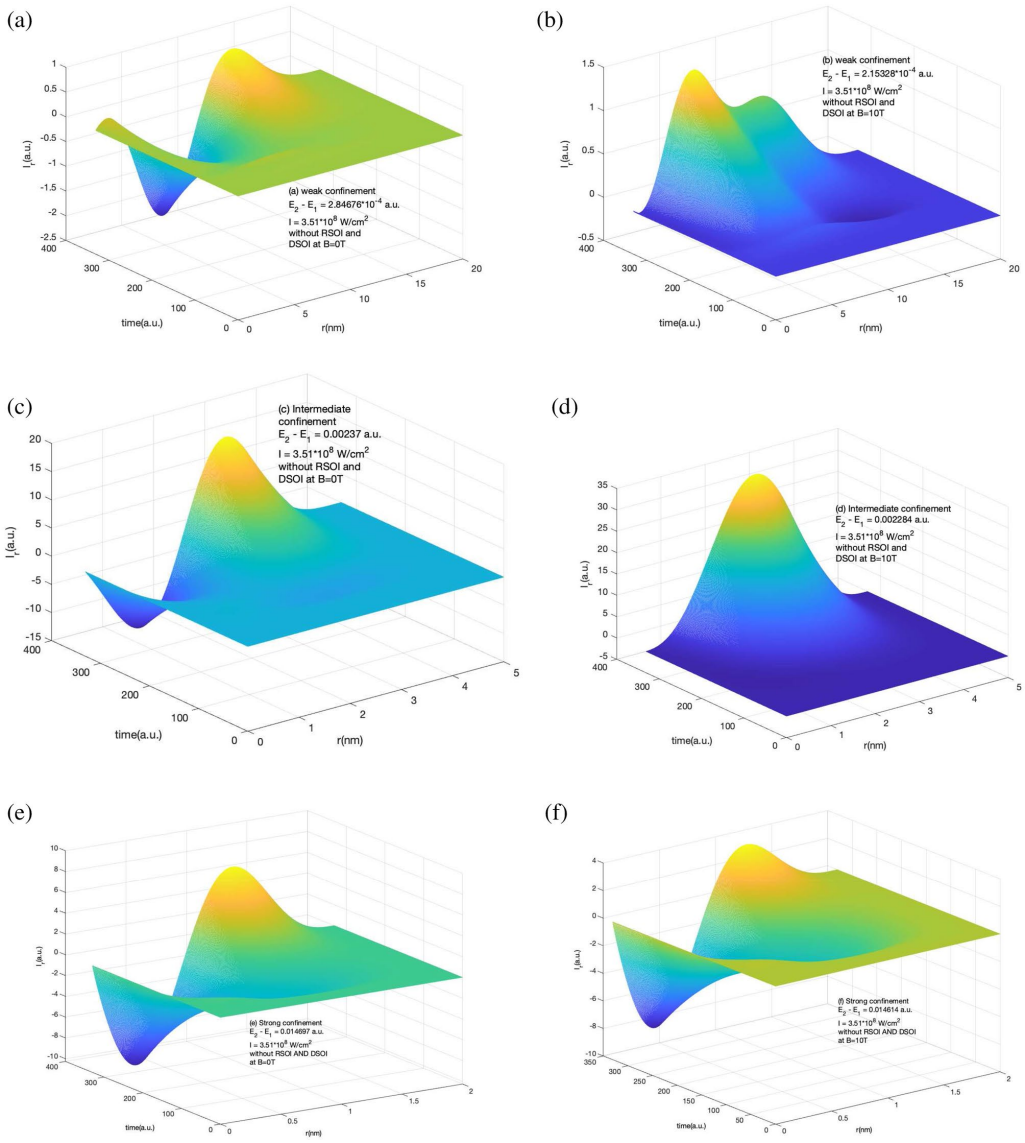
Variation of $I_r$ component variation in the absence of RSOI and DSOI with the time of the laser pulse (in a.u.) and *r* for a short laser pulse of $t_p$ = 4 fs, I = $10^{-4}$ a.u., $\hbar \omega = (E_2 - E_1)$ (**a**) for weak confinement region in the absence of magnetic field (**b**) for weak confinement region in the presence of magnetic field strength of 10 T (**c**) for intermediate confinement region in the absence of magnetic field (**d**) for intermediate confinement region in the presence of magnetic field strength of 10 T (**e**) for strong confinement region in the absence of magnetic field (**f**) for strong confinement region in the presence of magnetic field strength of 10 T.

In the absence of the SOI, as the current density changes with the duration of the laser exposed, therefore we expect the same response in the corresponding induced current also. This is shown below in Figs. 5 and 6.

Figures 5 and 6 shows $I_{\phi }$ and $I_r$ variation in the absence of RSOI and DSOI with the time of the laser pulse (in a.u.) and $\phi$ for $I_{\phi }$ and *r* for $I_r$ for a short laser pulse of 4 fs duration of intensity $10^{-4}$ a.u. of frequency equal to the difference of ground and first excited state for weak confinement region (a) and (b), for intermediate confinement (c) and (d), and for strong confinement (e) and (f) in the absence and presence of magnetic field respectively. Here we see that irrespective of confinement strength, the induced current has a variation with time this is so as the induced current density is evolving with time as shown in Figs. 1 and 2. In Fig. 5, we see that in weak and intermediate confinement i.e. Fig. 5a, b and c, d, the presence of the magnetic field increases the highest value of current but in a strong region the net amplitude of the induced current got decreased.

In contrast to the azimuthal component behavior without SOI in different confinement regions with and without magnetic field, the radial component of induced current has a different response. From Fig. 6 we see that in all three weak, intermediate, and strong confinement regions, the negative value of the $j_r$ decreases with the application of the magnetic field and the net amplitude of $I_r$ increases in weak and intermediate confinement regions and decreases in strong confinement region. The induce current evolves with time and reaches a maximum at a different time in different confinement regions.

**Figure 7:**
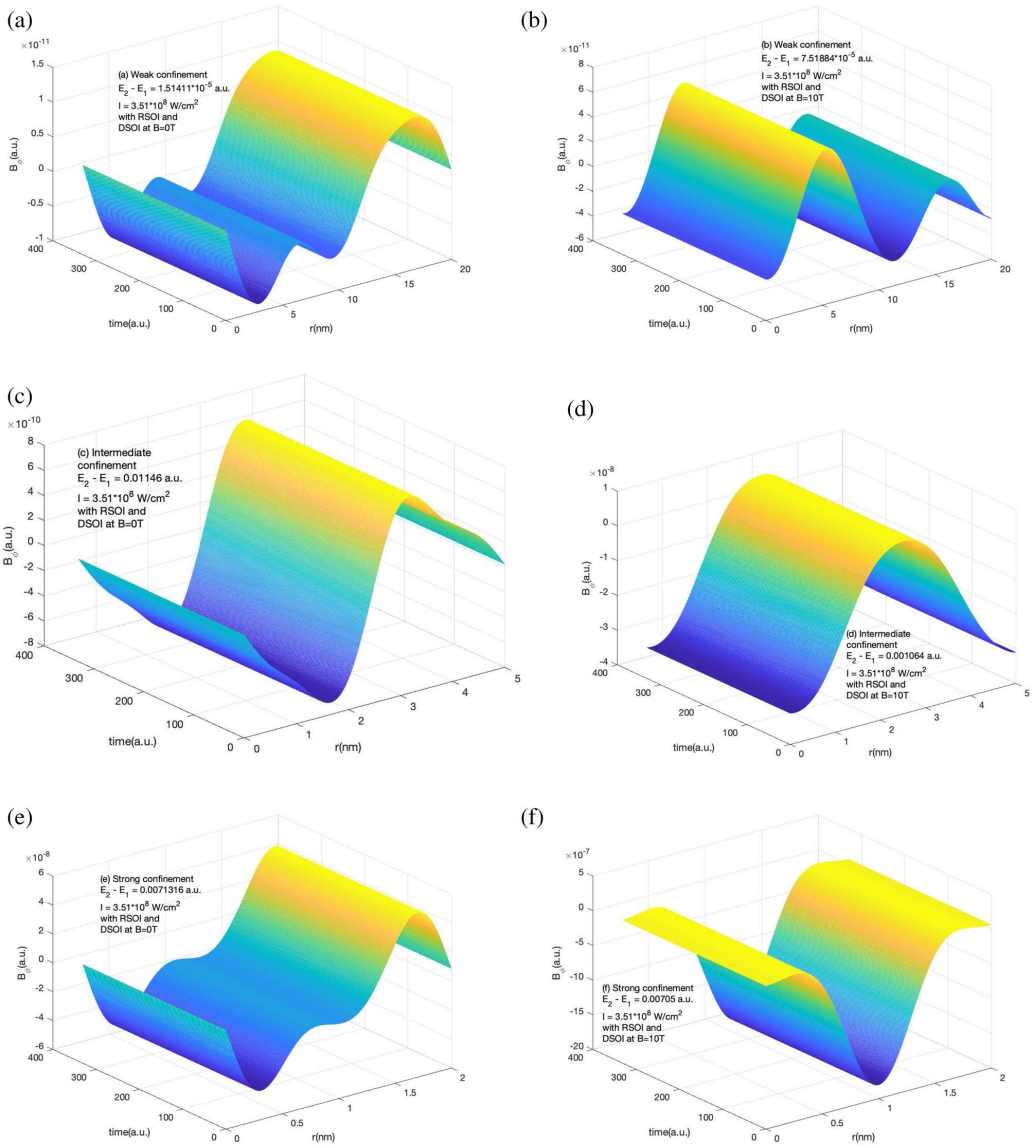
Variation of *B* in the presence of RSOI and DSOI with the time of the laser pulse(in a.u.) and *r* for a short laser pulse of $t_p$ = 4 fs, I = $10^{-4}$ a.u., $\hbar \omega = (E_2 - E_1)$ (**a**) for weak confinement region in the absence of magnetic field (**b**) for weak confinement region in the presence of magnetic field strength of 10 T (**c**) for intermediate confinement region in the absence of magnetic field (**d**) for intermediate confinement region in the presence of magnetic field strength of 10 T (**e**) for strong confinement region in the absence of magnetic field (**f**) for strong confinement region in the presence of magnetic field strength of 10 T.

Because the radial component of the magnetic field is zero, the azimuthal component contributes to the total magnetic field. Figure 7 represents the variation of the induced magnetic field in the presence of RSOI and DSOI with the time of the laser pulse(in a.u.) and *r* for a short laser pulse as explained in Fig. 1 and Supplementary information section. We observe positive and negative peaks along r and no variation along time as the current density remains constant with time (shown in Fig. 1) in the presence of SOI. The strength of the induced magnetic field increases as we increase the strength of the confinement by decreasing the confinement radius. The strength of the induced magnetic field increases on the application of the external magnetic field as the Zeeman energy term comes into play.

**Figure 8:**
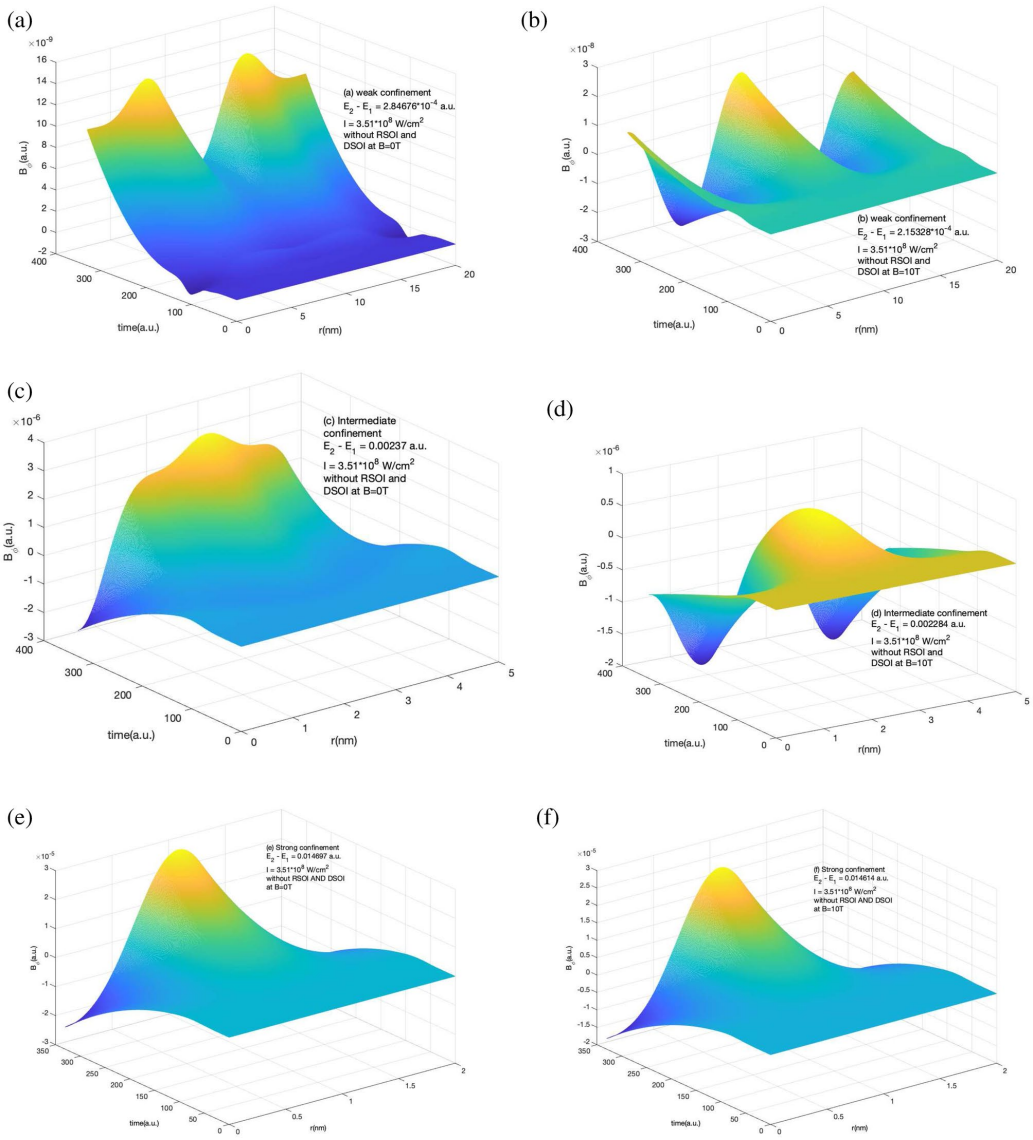
Variation of *B* in the absence of RSOI and DSOI with the time of the laser pulse(in a.u.) and *r* for a short laser pulse of $t_p$ = 4 fs, I=$10^{-4}$ a.u., $\hbar \omega = (E_2 - E_1)$ (**a**) for weak confinement region in the absence of magnetic field (**b**) for weak confinement region in the presence of magnetic field strength of 10 T (**c**) for intermediate confinement region in the absence of magnetic field (**d**) for intermediate confinement region in the presence of magnetic field strength of 10 T (**e**) for strong confinement region in the absence of magnetic field (**f**) for strong confinement region in the presence of magnetic field strength of 10 T.

The role of SOI is crucial as without SOI the current density evolves with time and hence induced current and magnetic field. Figure 8 shows the variation of the induced magnetic field in the absence of RSOI and DSOI with the time of the laser pulse (in a.u.) and r for a short laser pulse as explained in Fig. 1 and Supplementary information section. In weak confinement, the variation of $B_{\phi }$ is substantial. In intermediate confinement region, the Zeeman energy term with the competition to confinement effect suppresses the positive peak and the induce magnetic field becomes negative. However, in strong confinement region, the confinement effect dominates the Zeeman energy effect and hence small change is observed in $B_{\phi }$.

## Discussion

In the present study, we have studied a two-dimensional QR of $Si_{1-x}Ge_{x}$ where x is the Ge composition which is equal to $30\%$. The radial and azimuthal components of the induced current density have nearly compatible amplitudes so to study the net response of the system, it is a necessity to study them. The SOI lowers the magnitude of the induced current density, leading to lowering the strength of the induced current and induced magnetic field. We discovered that this quantum system's induced current and magnetic field are significant, making it a novel candidate for spintronic device fabrication. Both the radial and the azimuthal component of current density has positive and negative peaks. In case of weak confinement in presence of SOI, the strength of current density is also weak i.e. approx. $10^{-5}$ a.u. whereas in the case of strong confinement region, it is of the order of $10^{-3}$ a.u. (as shown in Supplementary Information section). The reason for this difference is that with an increase in confinement radius, the volume increases and the energy of quantum levels decreases, and the potential term behave as of harmonic potential so the role of the magnetic field is different in different confinement regions depending on the order of Zeeman energy term compared to the magnitude of energy levels. The presence of the SOI lowers the induced current density for both radial and azimuthal components which is a result from perturbation as SOI breaks the symmetry of the system and hence the degeneracy is lifted. This results in mixing of all states and we now have the total wavefunction as a linear combination of the individual states where the coefficient of mixing is important. The induced current density strength is dependent on the laser pulse also as it gives time-dependent mixing coefficients which can be altered using laser pulse duration $t_p$, intensity and the energy(frequency) of the laser pulse. The importance of Zeeman energy and SOI in modeling and tuning these induced fields and currents is critical. The laser pulse has a crucial effect as it gives freedom to manipulate the response of the system by suitable choosing the intensity, laser duration, and frequency of the beam. The laser period is more important in the absence of SOI as the induced current density, $I_{\phi }$, $I_r$, and $B_{\phi }$ shows variation with time.

We have calculated the values of induced current and magnetic field in the absence of SOI and magnetic field without the laser pulse. The values for the same are shown in Table 1. These values give us an insight about the strength of $I_{\phi }$ and $B_{\phi }$ and then with the effect of SOI, Zeeman energy and FLP, we will see how we can control these strengths (shown in Table 2).

**Table 1:** Induced azimuthal current ($I_{\phi }$) and induced magnetic field (*B*) values under different confinement regions without SOI and without short laser pulse in the absence of external magnetic field for few low lying states of the QR.

*n*	*l*	$B_{\phi }$ (Tesla) at confinement			$I_{\phi }$(mA) at confinement		
		Weak	Intermediate	Strong	Weak	Intermediate	Strong
0	0	0	0	0	0	0	0
0	− 1	− 7.95E−05	− 2.57E−03	− 4.00E−02	1.82E−01	5.26E−01	1.314072416
0	1	7.95E−05	2.57E−03	4.00E−02	− 1.82E−01	− 5.26E−01	− 1.314072416
0	− 2	− 9.34E−05	− 3.61E−03	− 5.63E−02	2.91E−01	9.17E−01	2.289569091
0	2	9.34E−05	3.61E−03	5.63E−02	− 2.91E−01	− 9.17E−01	− 2.289569091
1	0	0	0	0	0	0	0
1	− 1	− 8.94E−05	− 4.87E-03	− 7.61E-02	1.73E−01	6.70E−01	1.675314508
1	1	8.94E−05	4.87E−03	7.61E−02	− 1.73E−01	− 6.70E-01	− 1.675314508
1	− 2	− 1.11E−04	− 6.31E−03	− 9.86E−02	2.96E−01	1.150068548	2.874807981
1	2	1.11E−04	6.31E−03	9.86E−02	− 2.96E−01	− 1.150068548	− 2.874807981
2	0	0	0	0	0	0	0
2	− 1	− 1.16E−04	− 7.18E−03	− 1.12E−01	1.86E−01	0.763584955	1.909099692
2	1	1.16E−04	7.18E−03	1.12E−01	− 1.86E−01	− 0.763584955	-1.909099692
2	− 2	− 1.46E−04	− 8.99E−03	− 1.40E−01	3.25E−01	1.310828919	3.277065575
2	2	1.46E−04	8.99E−03	1.40E−01	− 3.25E−01	− 1.310828919	− 3.277065575

To summarize our results, we have produced the table given below to support the fact that the strength of induced current and magnetic field in our system is exceptional.

**Table 2:** Induced azimuthal current ($I_{\phi }$), induced radial current ($I_r$) and induced magnetic field (*B*) values under different confinement region with and without SOI at B = 0 T and B = 10 T in the presence of the short laser pulse.

Confinement	$I_{\phi }\,(mA)$		$I_{r}\,(mA)$		$B_{\phi } \,(T)$	
	B = 0 T	B = 10 T	B = 0 T	B = 10 T	B = 0 T	B = 10 T
Weak (SOI)	2.97E−03	0.02730661293	4.24E−02	0.04246343351	2.80E−06	1.23E−05
Intermediate (SOI)	0.01390956	0.4797367901	0.14704392	0.4498629871	1.80E−04	7.34E−03
Strong (SOI)	0.1126012	4.53915308	0.66765888	4.14107472	1.27E−02	4.24E−01
Weak	3.32372248	10.08972988	13.82477792	9.54063344	3.31E−03	5.85E−03
Intermediate	79.25667288	60.53904164	113.5516866	228.9632801	8.53E−01	4.57E−01
Strong	119.4159571	100.1151841	6.82E+01	5.56E+01	6.63E+00	5.48E+00

From Table 2, it is clear that with an increase in the magnetic field in presence of SOI in all confinement strengths region, the amount of induced current and magnetic field increases. This is due to the interplay of Zeeman energy in the system. On the contrary, in the absence of the SOI, the azimuthal current ($I_{\phi }$) and induced magnetic field in the intermediate and strong confinement region suffers a decrease in the induced amplitude and $I_{r}$ gains the amplitude. However, in the weak confinement, $I_{\phi }$, $I_{r}$, and induced magnetic field, gains in the amplitude when external magnetic field is applied.

As it is visible from Table 2 that intermediate confinement induces maximum radial current i.e., $I_{r}$ without SOI. The reason behind this is that both the parabolic and inverse square potential comes into play which are antagonistic. In the strong confinement, we have dominating inverse square potential and in the weak confinement, we have dominant parabolic potential in picture. The induced magnetic field strength is largest in strong confinement region. The SOI lowers the induced strength as it breaks the symmetry of the system which lift the degeneracy of the system.

On comparing the Tables 1 and 2, we can see the change in induced current and fields which is revolutionary in itself. This study has relevance in spintronic modeling where the role of external magnetic field and SOI is sought as here we can harness large induced currents and magnetic fields.

## Conclusion

In the present study, we have studied a two-dimensional quantum ring of $Si_{1-x}Ge_{x}$ where x is the Ge composition equal to $30\%$. The radial and the azimuthal components of the induced current density have nearly compatible amplitudes, so to study the net response of the system, it is necessary to study them. The SOI lowers the magnitude of induced current density, lowering the strength of induced current and induced magnetic field. This quantum system’s induced current and magnetic field are significant, making it a novel candidate for spintronic device fabrication. The importance of Zeeman energy and SOI in modeling and tuning these induced fields and currents is critical. The laser pulse has a crucial effect as it gives freedom to manipulate the system’s response by choosing the beam’s intensity, laser duration, and frequency. The laser period is more critical in the absence of SOI as the induced current density, $I_{\phi }$, $I_r$, and $B_{\phi }$ shows variation with time. There is a giant leap in the magnitude of the induced currents and magnetic fields, as listed in the tables in the discussion section. This study is relevant in spintronic modeling where the role of external magnetic field and SOI is sought, as we can harness large induced currents and magnetic fields here.

## Data Availability

This manuscript has no associated data added to any data repository. [Authors’ comment: The datasets generated during and/or analyzed during the current study are available from the corresponding author on reasonable request].
